# Pediatric Patients With Hemiplegia: A Systematic Review of a Randomized Controlled Trial

**DOI:** 10.7759/cureus.34074

**Published:** 2023-01-23

**Authors:** Ashish Varma, Nadeem R Khan, Anuj Varma, Nidhi S Sharma, Jayant D Vagha, Waqar M Naqvi, Smruti Besekar

**Affiliations:** 1 Department of Pediatrics, Jawaharlal Nehru Medical College, Datta Meghe Institute of Higher Education and Research, Wardha, IND; 2 Department of Community Medicine, Sukh Sagar Medical College, Jabalpur, IND; 3 Department of Medicine, Jawaharlal Nehru Medical College, Datta Meghe Institute of Higher Education and Research, Wardha, IND; 4 Department of Physiotherapy, Ojas College of Physiotherapy, Jalna, IND; 5 Department of Pediatrics, Jawaharlal Nehru Medical College, Datta Meghe Institute of of Higher Education and Research, Wardha, IND; 6 Department of Internal Medicine, Ravi Nair Physiotherapy College, Datta Meghe Institute of of Higher Education and Research, Wardha, IND; 7 Directorate of Research, NKP Salve Institute of Medical Sciences and Research Center, Nagpur, IND; 8 Department of Pharmacology, Datta Meghe Institute of of Higher Education and Research, Wardha, IND

**Keywords:** pediatric, randomized controlled trial, systematic review, kinesio taping, hemiplegia

## Abstract

Hemiplegia is the medical term for paralysis of one side of the body. It results in muscular wasting on the affected side, impairs gait, reduces motor abilities, and causes instability and a loss of grasping capacity. The patient's quality of life is impacted by hemiplegia because it impairs brain and spinal cord functions. Consequently, a range of therapeutic options, including physical therapy, medical health management, and other multidisciplinary care, are accessible. The effects of treatments on juvenile patients with hemiplegia who are participating in a randomized controlled trial (RCT) are examined in this systematic review. Using the Boolean operator "AND," the research process entailed searching for keywords like "Hemiplegia" and "Pediatrics." Based on the inclusion and exclusion criteria, a total of six RCTs were included in the study. According to the study's findings, hemiplegic patients benefited from Kinesio taping (KT), botulinum toxin type-A (BoNT-A), hyaluronic acid injections, and bimanual treatment.

## Introduction and background

Hemiplegia is a nonprogressive disorder that results in paralysis on one side of the body and is caused by brain or spinal cord trauma. Depending on the location and severity of the injury, the degree of hemiplegic symptoms varies. Congenital hemiplegia refers to the onset of hemiplegia before, during, or within the first two years of life. Acquired hemiplegia is a term used to describe hemiplegia occurring later in life [1].

Hemiplegia is caused by conditions such as stroke, brain infections (by bacteria, fungi, or viruses), brain trauma, brain tumors, and rare mutation in genes (alternating hemiplegia). Hemiplegia is a more general name for cerebral palsy (CP), which develops before birth and manifests in the first few years of life. Other hemiplegia types include alternating, facial, spinal, contralateral, spastic, and spastic hemiplegia [1,2].

Depending on the degree, hemiplegia symptoms may include muscle stiffness or weakness on one side, spasticity or permanently clenched muscles, poor fine motor skills, difficulty walking, unsteadiness, and difficulty grasping objects. Additionally, hemiplegic children are less active and require more developmental years than healthy children do. Additionally, they are permitted to play with just one hand or to hold one hand in a fist. If brain injury results in hemiplegia, brain damage may result in symptoms other than hemiplegia, such as memory loss, difficulty focusing, speech problems, behavioral disorders, and seizures. Various treatment modalities are available based on severity such as physiotherapy, multidisciplinary rehabilitation such as physical therapy, mental health therapist, and other medical management [1]. This review was conducted to oversee the different interventional therapies and their effects on hemiplegia pediatric patients.

## Review

Research methodology

**Figure 1:**
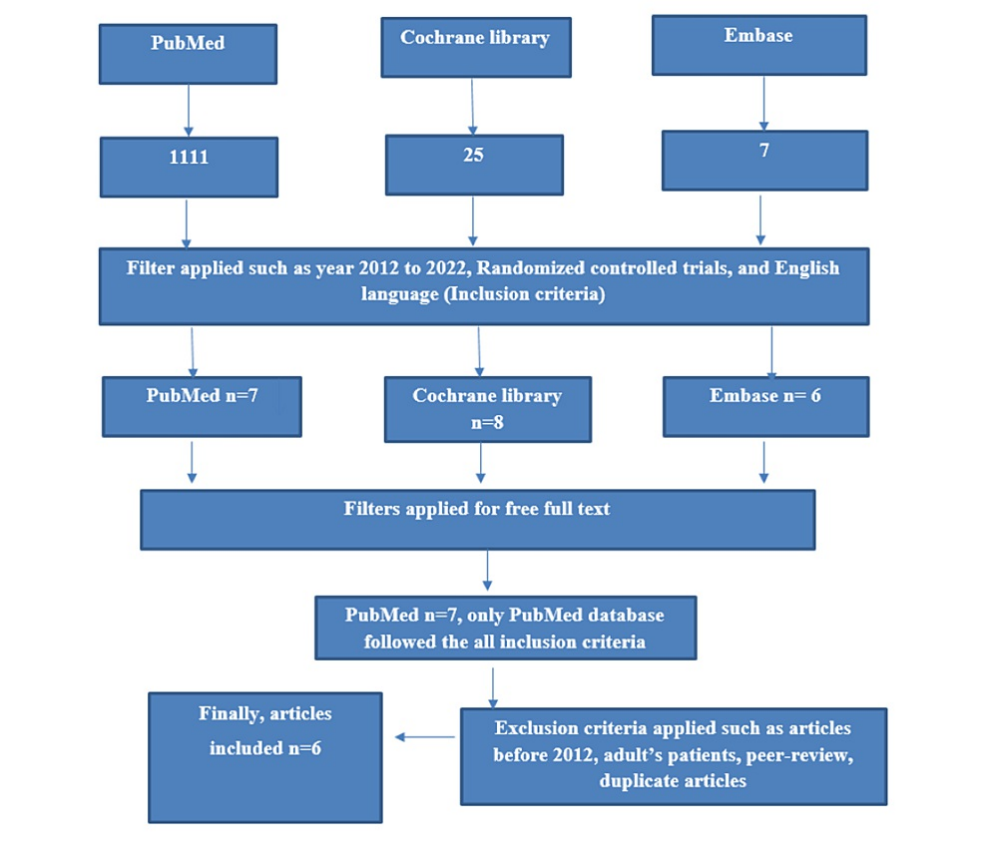
Search strategy for hemiplegia and pediatrics from the databases. Figure credits: All authors.

The updated Preferred Reporting Items for Systematic Reviews and Meta-Analyses literature search extension (PRISMA-S) statement for reporting systematic reviews was used to conduct this systematic review [3]. We searched the PubMed, Cochrane Library, and Embase databases for randomized controlled trials (RCTs) published between 2012 and July 2022. The search terms from the two search topics were combined to search the database using the Boolean operator "AND." The many investigations that have been carried out on the pediatric population are quantified using the text words "hemiplegia," "pediatrics," and "cerebral palsy." English language studies that examined various pharmacological and interventional treatments used in hemiplegic patients were included in this study. The articles included had the following characteristics: free complete abstracts, RCTs, and publication years between 2012 and 2022. Peer reviews, observational studies, case reports, trials conducted before 2012, trials involving adult patients, and publications without open full text or access were omitted. The RCT inclusion criteria and selection process are shown in Figure 1.

Results

From the database searches, 1,135 published papers were identified. After examining the titles and abstracts, 32 RCTs about childhood hemiplegia were selected for free full-text review. Thus, using inclusion parameters, six RCTs were collected for a thorough review. However, the comparison among the different interventions on pediatric patients with hemiplegia and CP was evaluated through this study. Table 1 depicts the details about the studies involved, author’s name, country, database, year of publication, and journals.

**Table 1:** Information regarding the study’s author name, year of publication, journal name, and place where the study conducted.

Serial no.	Author name	Journal name	Database	Year	Country
1	Huang et al. [3]	European Journal of Physical and Rehabilitation Medicine	PubMed	2016	Taiwan
2	Hastings-Ison et al.[4]	Development Medicine and Child Neurology	PubMed	2016	Australia and New Zealand
3	Huang et al.[2]	Walter Kluwer Medicine	PubMed	2016	Taiwan
4	Friel et al. [5]	SAGE Journals	PubMed	2016	Columbia
5	Hastings-Ison et al. [6]	Wiley Journal	PubMed	2013	Australia
6	Hoare et al. [7]	Wiley Journal	PubMed	2012	Australia

The comparison among the intervention groups and study outcome and the inclusion criteria of the study are shown in Table 2.

**Table 2:** Information regarding the study purpose, inclusion criteria, outcomes, and study groups. KT, kinesio taping; HSP, hemiplegic shoulder pain; AROM, active range of motion; BoNT-A, botulinum toxin type-A; HA, hyaluronic acid; USCP, unilateral spastic cerebral palsy; COPM, Canadian Occupational Performance Measure; CP, cerebral palsy; GMFCS, Gross Motor Function Classification System; SEM, standard error of the mean; mCIMT, modified constraint-induced movement therapy

Serial no.	Author’s name	Inclusion	Purpose	Study groups	Study outcome
1	Huang et al. [3]	*n* = 44, subacute stroke hemiplegia	The effects of KT on HSP, upper extremity functional outcomes, and reduction of soft tissue damage were assessed in subacute stroke patients with hemiplegic shoulders during rehabilitation.	Randomly allocated to the therapeutic KT and the control group (sham)	The findings show that kinesiology tapping helps patients with HSP after stroke feel better by decreasing shoulder discomfort and subluxation, and boosting muscular activation and AROM.
2	Hastings-Ison et al. [4]	*n* = 42, ambulant children with spastic equinus, secondary to CP	For spastic equinus on passive dorsiflexion, the frequency of BoNT-A injections was evaluated and compared at 12 months versus four months.	The calf muscle got 12 monthly/four monthly injections of BoNT-A throughout a 26-month period after being randomly assigned. Additionally, 6 U/kg of Botox was administered into the gastrocnemius muscles of the affected limbs under mask anesthesia.	Passive dorsiflexion and secondary outcome indicators did not differ significantly between the injection regimens administered every 12 and every four months. For spastic equinus, a 12-month cycle of BoNT-A injections is recommended.
3	Huang et al.[2]	*n* = 26, subacute stroke patients	Motor function and pain alleviation in subacute stroke patients with HSP and damage affected by HA injection were assessed.	The experimental group (n = 16) received ultrasound-guided subacromial HA injections once weekly for three weeks as opposed to the control group (n = 10) received 0.9% sodium chloride injections once weekly for three weeks along with conventional rehabilitation.	Subacute stroke patients with HSP and damage may experience improvements in shoulder discomfort and abduction after receiving a subacromial HA injection.
4	Friel et al. [5]	*n* = 20, unilateral spastic CP	Compared with unstructured practice, systematic skill training results in more motor map plasticity.	The impact of bimanual therapy alternates between unstructured play-like hand use and systematic skill training on children with USCP's manual skills.	Bimanual hand use and dexterity significantly increased in both groups. The size of the motor map of the distressed hand and the magnitude of motor-evoked potentials increased only in the structured skill group. The majority of the children who demonstrated the greatest functional gains (COPM) also had the greatest changes in map size.
5	Hastings-Ison et al. [6]	*n* = 34 with spastic CP - GMFCS level of I to III	Assess the ability of an instrumented measure to reduce measurement error in children with CP to less than 5°, as well as its agreement and reliability in both conscious and anesthetized participants. Finally, assess how well the method compares to other forms that have been previously reported.	The children were randomly assigned to the hemiplegic, diplegia, and quadriplegic groups and measured SEM with the previously reported dorsiflexion measures in conscious CP patients. The children were measured when conscious and under mask anesthesia.	The SEM ranged from 3.9° and 6.7° in anesthetized and conscious patients, respectively, as compared to the previously reported dorsiflexion, which was between 6.5° and 7.8° in conscious CP. This method is not clinically advised because it does not produce better results than trained assessors using standard goniometry, as documented in the literature.
6	Hoare et al. [7]	*n *= 34, unilateral CP	This study investigated whether mCIMT produces improvements that are sufficiently superior to bimanual occupational therapy in young children with unilateral CP after BoNT-A injection (BOT).	The experimental group received mCIMT and BoNT-A. In the comparison group, BoNT-A and BOT were given.	The use of mCIMT did not show any significant results in children with unilateral CP as compared with BoNT-A.

Discussion

This systematic review demonstrates the different methods related to pharmacological and physical therapies in pediatric hemiplegic patients. However, hemiplegia affects half of the body's function, and it is crucial to understand the different management alternatives for a better prognosis for patients.

It remains difficult to effectively treat upper limb (UL) dysfunction in patients with hemiplegia. Hemiplegic shoulder pain (HSP), which occurs in 17% to 72% of stroke survivors, is one of the most frequent consequences. HSP is always linked to the slower recovery of UL functional ability, interference with rehabilitation training, and a lower quality of life. Appropriate placement of shoulder slings that provide support, functional electrical stimulation, physical therapy, acupuncture, and steroid or Botox injections can reduce HSP discomfort and spasticity [3].

Currently, kinesio taping (KT) is the most frequently used clinical treatment for musculoskeletal disorders. KT can be helpful in many conditions, such as enabling blood circulation, providing mechanical support and proprioceptive feedback, improving joint range of motion, and stimulating muscles. Hence, KT is considered an effective treatment means for HSP and spasticity. The positive side of this trial was the completion of the study by the enrolled patients. At baseline, no evident differences were observed between the therapeutic KT and standard taping groups. The first-day pain intensity, subluxation severity, and muscle activity improved in the taping group, while the control group experienced no appreciable changes. Hence, the KT group was effective in improving all functions [3]. According to earlier research, KT increases blood flow and lymphatic discharge, which reduces edema and provides pain relief. It is still a riddle about KT's pain-relieving function remains baffled [8,9]. Other studies have indicated that there are significant variations between KT and traditional therapy in terms of muscular spasticity [10], whereas other studies have stated that there was no statistically significant change in muscle tone [11]. Therefore, KT is safe and efficient. The taping technique appeared to differ in various studies, including variations in the target muscle, direction, tension, and concurrent treatment [12]. Another study found that there was an increase in concentric elbow peak torque in a population of healthy volunteers when KT was used in comparison to a placebo [13].

Similarly, elastic taping is known to improve mobility and independence by strengthening the muscles, while elastic tape keeps the agonists, synergists, and antagonist muscles coordinated [14]. Elastic tapping was employed for 30 minutes in the RCT of 11 patients who had hemiparesis after having a stroke to improve their aberrant gait length and speed. Therefore, the study demonstrated that when collate to the pre-application value, gait pace and step extent on the healthy side improved after half an hour of elastic taping. In conclusion, poststroke patients with weak ankle dorsiflexors showed increased gait speed and step length [15].

Another study included two centers to compare the frequencies of botulinum toxin type-A (BoNT-A) injections for spastic equinus. Equinus, which comprises contraction of the gastrocnemius or the muscle-tendon complex, is the most prevalent spastic abnormality in CP (triceps surae) [16]. It may be paired with an equino valgus deformity of the foot when pronation is present due to tense peroneal muscles or an equinovarus deformity when the tibialis posterior muscle is overactive or tense. Injections into the gastrocnemius muscles of both lower limbs were administered to children with spastic diplegia at a dosage of 6 to 12 U/kg. Under mask anesthesia and electrical stimulation, 6 U/kg of fixed-dose botox was administered intravenously into the gastrocnemius at one site in the lateral belly and two sites in the medial belly of the calf muscle [5]. The trial findings support the recommendation of a 12-unit monthly injection as therapy. In the end, 12-monthly injections are recommended for the treatment of spastic equinus in children with hemiplegia and diplegia [5]. Based on research, children with hemiplegia did not profit from an increase in the injection frequency. A transition to a permanent contracture that is unresponsive to more frequent injections occurs in early childhood. A similar study by Kanovský et al. showed that for the group as a whole, BoNT-A injections every four months did not offer any benefit over injections every 12 months for spastic equinus [17]. Nevertheless, over the two-year study period, passive dorsiflexion was maintained in both therapy groups. This might imply a benefit compared to standard natural history, but it is impossible to prove this without a control group [17]. The loss of passive dorsiflexion in hemiplegic children who received injections every four months was 9.0° during the 26 months, compared to 8.1° for those who received injections every year. Children with hemiplegia who receive injections three times per week develop contractures. When a fixed contracture form, the study advised stopping BoNT-A injections and referring patients as soon as possible to surgically extend the contracture [5].

Patients with acute stroke and flaccid shoulders frequently experience HSP. HSP lessens the length of hospital stays, quality of life, and functional recovery following a stroke. HSP in stroke patients has been treated with a variety of interventions, including physical modalities, exercise, medicine, and localized injections [18].

In another study, HSP is associated with rotator cuff injuries and can be treated with steroids or hyaluronic acid to relieve pain. In the interventional group, the subdeltoid bursa was injected with 2.5 mL of sodium hyaluronate under ultrasound guidance, while the subdeltoid bursa was injected with 2.5 mL of 0.9% sodium chloride for the control group and was also enrolled in the inpatient rehabilitation program. No negative outcomes, such as tendon ruptures and tissue deterioration, occurred, and rotator cuff injuries patients received superior care. The RCT did not include a longer follow-up duration for patients with stroke who received HA injections. As the sample size was small and only included patients from one center, this study did not record the specifics of the physical modalities used for HSP pain management [2].

Patients with unilateral spastic CP (USCP) have weakness and motor dysfunction due to injury to the developing brain. Improving hand function is a key priority for the majority of children with USCP and can be addressed by intensive bimanual therapy or hand-arm bimanual intensive therapy (HABIT), which assesses the functional changes in the brain. In this study, the kids were divided into two groups for bimanual skill training that was both structured and unstructured. The study demonstrated that skill training with the aid of a motor map enhanced the positive aspects, such as the strength, functional performance, and size of the motor map of the affected hand. According to this study, neuroplasticity exhibits dichotomy. Although hand performance increased, there was no evidence of motor cortical plasticity in the unstructured practice group's transcranial magnetic stimulation (TMS) map. The 90-hour high dose may have eradicated the group differences. Variations between the groups could have been caused by lower dosages. Although the study found a connection between cortical plasticity and functional improvements, it is possible that at lower doses, the association with plasticity for the organized skill group might be stronger than that of the unstructured group. Although the groups were matched for age and Jebsen-Taylor test of hand function (JTTHF) baseline, the distribution of CST projection patterns showed differences between the groups, and the study only examined M1 plasticity. The study's limited sample size limits its generalizability [5].

The terms agreement and reliability are combined under the phrase *reproducibility*, which refers to the degree to which results from repeated measurements are consistent. An attribute of the measurement tool itself is agreement. Reliability is the capacity to distinguish between research components (participants), notwithstanding the measurement inaccuracy caused by participant variability.

The subtalar joint exhibited intricate and triplanar movements. Despite efforts to standardize coronal and transverse joint mobility while assessing passive dorsiflexion range with the knee extended (PADKE) in the sagittal plane in this study of young children, the standard deviation for assessments performed under anesthesia decreased for one assessor alone. When comparing the repeatability of PADKE in ambulant children with CP to previously published measures, the instrumented technique was successful in standardizing the applied torque. Reproducibility was enhanced when one of the two assessors was under anesthesia. The methodological shortcomings included time and budget limitations. Scientists found it challenging to implement dorsiflexion strength, hold the knee and footplate steady, and take photos. Adopting an instrumented procedure, the outcomes were comparable to those of the existing methods. The universal goniometer is so straightforward that it is not recommended professionally during conscious examinations. It should be noted that the suggested 5° minimal clinically important difference (MCID) will not be implemented [6].

In addition to receiving BoNT-A, unilateral CP children who received occupational therapy experienced improvements in their UL outcomes CP. In this trial, BoNT-A was merged with constraint-induced movement therapy (CIMT), a group of procedures meant to minimize the effects of stroke on UL function in some stroke survivors. The *Learned- Nonuse* behavioral approach to neurorehabilitation is applied. This RCT revealed no proof that mCIMT had a better influence on nearly all outcomes than BOT. This was true even when statistical methods were employed to demonstrate superiority over the strongest evidence of chance. The key hypothesis that more intense practice would make mCIMT superior was not supported. There were several issues with the trial, including the single trial RCT; the small sample size; the children, families, and interventionists' understanding of group assignment; and the absence of a real comparison group that would have received BoNT-A alone without movement-based treatment [7].

Children with hemiplegic CP have abnormally developing motor systems; impaired static, sensory, and visual skills; and vicious cycle limitations. They also have excessive thumb abduction and flexion and limited active wrist extension. This study evaluated the effectiveness of robot-assisted therapy, bimanual therapy, virtual reality, and CIMT. Consequently, the study often had brief results and tended to be smaller. Modified CIMT and action observation therapy (AOT) are two interventions investigated for newborns. The use of elastic taping is another strategy that might be suitable for a baby with a thumb-in-palm malformation; however, it has to be evaluated. It has been proposed that the tape's increased mechanical stimulation of the skin during movement can influence the continuing muscle activity [19].

A variety of methods have been devised with varying degrees of success to enhance hand function in children with HCP [20]. Current treatment methods essentially involve having the child actively practice the desired movements repeatedly (often with shaping, which breaks the objective down into smaller, more manageable steps as progress is made) [21]. The majority of exercises reviewed the concepts of motor training and neuroplasticity and are tailored to the child's age and cognitive capabilities. Methods usually are game-oriented, analytical, or goal-focused, for instance, or use of virtual reality, and robotic mechanics in daily life activities, represent alternative modalities for UL therapy [22], rather than fundamentally novel ideas; hence, new research integrating noninvasive brain stimulation with occupational therapy techniques for older children with hemiplegia is needed [23].

Robotic treatment is a novel approach that has demonstrated promise in enhancing motor function, enhancing the quality of life, and lessening the burden on caregivers [24]. Motion 2 and NJIT-RAVR systems are the two most widely utilized touch robot systems. According to preliminary results, InMotion2 led to greater gains in kinematic variables, muscular tone, and strength, as well as in standardized evaluation measures. Despite showing promise in enhancing reaching kinematics, the New Jersey Institute of Technology-Robot-Assisted Virtual Rehabilitation (NJIT-RAVR) system did not result in comparable improvements in standardized clinical evaluation measures. One study used a noncontact robot and a randomized crossover design to treat children with CP [24]. When a child's movement did not adhere to a preestablished condition, the robot (CosmoBot, Anthrotronix Inc., Silver Spring, MD, USA) provided accurate feedback. It is interesting to note that robotic feedback therapy outperformed traditional therapy in helping children achieve their goal range of motion [25].

Various treatments include applying a cast, tape, or external device to the afflicted joints, as well as hand splits or orthotics [26]. The use of splints in young children is typically challenging because they would want to take them off and make a well-fitting replacement, as small hands typically make splints difficult [27]. A technique called AOT aims to engage a frontoparietal matrix of neurons that is active throughout action surveillance and action presentation [28]. In animal research, Rizzolatti and Craighero first discovered mirror neurons, which were labeled as they lit up when the animal either engaged in a motor function or viewed another animal engaging in the same function [28]. Adults with stroke responded well to treatment that included frequent action observation and imitation [29].

Pediatric hemiplegia rehabilitation focuses on conquering developmental disuse with the help of constraint-induced treatment (CIT) and low-frequency repeated TMS (rTMS), a noninvasive method of activating specific brain regions. The planned study is significant because pediatric hemiplegia has received scant attention in studies on rehabilitation-induced brain reconfiguration. It utilizes a technique that has never been employed before, thus making it revolutionary [30]. The results of the study confirm the notion that virtual reality (VR) therapy may be a promising and entertaining tool for pediatric rehabilitation in congenital hemiplegia [31].

One of the conditions known as hemiplegic migraine (HM) was studied in children and examined clinical manifestations, including sporadic and familial HM [32]. The most common treatment for such conditions is analgesics, calcium channel blockers, beta-blockers, tricyclic antidepressants, and antidepressants [33]. There is no specific treatment for alternating hemiplegia (AH), but therapies mainly focused on reducing the frequency and severity of the episodes. Drugs such as flunarizine, benzodiazepines, chloral hydrate, melatonin, and rectal diazepam are usually prescribed [34,35].

There is a dire need for an early interventional program for treating developmental delays and disabilities at early stages of life that includes the cognitive, adaptive, community, physical, and communication development that improves the further life of young children [36].

The drawbacks of this study were that the included RCTs were from less than two sites and had a few participants; therefore, there might be chances of the investigator's bias. Second, every single RCT had a unique design that was put into practice in various contexts, over various time frames, and with various control treatments. These variations may have developed because of the various interventions performed. Third, there is a possibility that the variety of measurements led to comparable heterogeneity. Finally, only less number of RCTs were enlisted; hence, it does not conclude the potential benefits of interventional therapy compared to conventional therapy in the management of hemiplegic patients.

## Conclusions

This study stated that there were differences in the outcome of each RCT. Therapies like KT, BoNT-A, hyaluronic acid injections, bimanual therapy, hand use, and systematic skill training showed better improvements in hemiplegic pediatric patients. Therefore, no significant outcome can be found as the intervention treatment group slightly showed a beneficial effect and improvements as compared to the control group and vice versa. Hence, more studies are needed for investigating and analyzing the importance of experimental tools in the RCT.
